# Loss-of-function tolerance of enhancers in the human genome

**DOI:** 10.1371/journal.pgen.1008663

**Published:** 2020-04-03

**Authors:** Duo Xu, Omer Gokcumen, Ekta Khurana

**Affiliations:** 1 Institute for Computational Biomedicine, Weill Cornell Medicine, New York, New York, United States of America; 2 Department of Physiology and Biophysics, Weill Cornell Medicine, New York, New York, United States of America; 3 Englander Institute for Precision Medicine, New York Presbyterian Hospital-Weill Cornell Medicine, New York, New York, United States of America; 4 Meyer Cancer Center, Weill Cornell Medicine, New York, New York, United States of America; 5 Department of Biological Sciences, University at Buffalo, The State University of New York, Buffalo, New York, United States of America; UCSF, UNITED STATES

## Abstract

Previous studies have surveyed the potential impact of loss-of-function (LoF) variants and identified LoF-tolerant protein-coding genes. However, the tolerance of human genomes to losing enhancers has not yet been evaluated. Here we present the catalog of LoF-tolerant enhancers using structural variants from whole-genome sequences. Using a conservative approach, we estimate that individual human genomes possess at least 28 LoF-tolerant enhancers on average. We assessed the properties of LoF-tolerant enhancers in a unified regulatory network constructed by integrating tissue-specific enhancers and gene-gene interactions. We find that LoF-tolerant enhancers tend to be more tissue-specific and regulate fewer and more dispensable genes relative to other enhancers. They are enriched in immune-related cells while enhancers with low LoF-tolerance are enriched in kidney and brain/neuronal stem cells. We developed a supervised learning approach to predict the LoF-tolerance of all enhancers, which achieved an area under the receiver operating characteristics curve (AUROC) of 98%. We predict 3,519 more enhancers would be likely tolerant to LoF and 129 enhancers that would have low LoF-tolerance. Our predictions are supported by a known set of disease enhancers and novel deletions from PacBio sequencing. The LoF-tolerance scores provided here will serve as an important reference for disease studies.

## Author summary

Enhancers are elements where transcription factors bind and regulate the expression of protein-coding genes. Although multiple previous studies have focused on which genes can tolerate loss-of-function (LoF), none has systematically evaluated the tolerance of all enhancers in the human genome to LoF. Individual studies have shown a broad range of phenotypic effects of enhancer LoF. The phenotypic effects of enhancer LoF likely fall into a spectrum where deletion of LoF-tolerant enhancers would not elicit substantial phenotypic impact, while some enhancers are likely to cause fitness defects when deleted. Here we report a systematic computational approach that uses machine learning and properties of enhancers in a unified human regulatory network with tissue-specific annotations to predict the LoF-tolerance of all enhancers identified in the human genome. The LoF-tolerance scores of enhancers provided in this study can significantly facilitate the interpretation and prioritization of non-coding sequence variants for disease and functional studies.

## Introduction

Loss-of-function (LoF) variants in genes are defined as those which impair or eliminate the function of the encoded protein. Despite their protein-coding disruption, it has been shown that some LoF variants can be tolerated in healthy individuals [1–4]. Genes harboring homozygous LoF variants are called LoF-tolerant genes. Multiple studies have shown the average number of LoF variants ranges from 100~200 per individual [5–7]. In addition, MacArthur et al estimated that on average there are 20 LoF-tolerant genes per human genome [5]. Such lists of LoF variants have greatly aided gene prioritization in disease studies by providing functional references for variants [8–12]. It also enabled estimations of gene indispensability by providing a confident set of LoF variants and LoF-tolerant genes in human genomes [5, 13].

However, in stark contrast to protein-coding genes, our knowledge about the dispensability of non-coding regulatory elements is limited. The atlas of cell- and tissue-specific regulatory elements developed by large-scale efforts, such as ENCODE [14, 15], Roadmap Epigenomics Mapping Consortium [16], FANTOM [17] and the availability of thousands of whole-genomes makes this an opportune time to ask the same questions that were asked for protein-coding genes and to identify the non-coding elements that can tolerate homozygous LoF.

Enhancers can act redundantly in groups to regulate gene expression instead of stand-alone units.

Such ‘shadow’ enhancers are defined as the ones that have similar functions to the proximal primary enhancers but locate at distal locations [18]. It has been observed in Drosophila that while deleting one enhancer may not cause phenotypic changes, deleting both the primary and the secondary enhancers leads to fitness defects [18–20]. It has also been shown that deletion of some individual enhancers in mice did not significantly affect their fitness, but deletion of pairs of enhancers regulating the same gene led to abnormal limb development, indicating the redundancy of enhancers leads to robustness in gene expression [21]. Thus, it is thought that the phenotypic effects stemming from the loss of a single enhancer in humans may be mitigated by the activity of another enhancer, whose function is redundant to the deleted one, and is therefore only apparent if both enhancers are deleted [22]. These studies may lead one to the interpretation that loss of an individual enhancer is not likely to produce strong phenotypic effects. However, it has been shown that alterations at single enhancers are linked to rare Mendelian diseases [23–26]. Thus, based on our current understanding, the phenotypic effects of enhancer LoF likely fall into a spectrum where deletion of LoF-tolerant enhancers would not elicit substantial phenotypic impact, while some enhancers are likely to cause fitness defects even when single enhancers exhibit LoF. A prioritization scheme based on LoF-tolerance scores of enhancers can help identify causal sequence variants at enhancers in disease studies. Mutations (single nucleotide variants (SNVs), short insertions and deletions (indels) and structural variants (SVs)) at enhancers with high LoF-tolerance are less likely to produce fitness defects while variants at enhancers with low tolerance to LoF are more likely to be disease-causing. Such prioritization scheme will not only help understand the causal variants of Mendelian diseases, it will also provide insights for the many non-coding susceptibility loci found by genome-wide association studies (GWAS) [27–31] of which the potential causations beneath the associations are still unknown.

Here we report a systematic computational approach that uses machine learning to predict the LoF-tolerance of enhancers identified in the human genome using ENCODE and Roadmap Epigenomics Consortium data [14–16]. We built an integrated regulatory network, MegaNet, in which the nodes consist of enhancers and genes. The edges between enhancers and genes correspond to tissue-specific regulation and those between genes include protein-protein [32], metabolic [33], phosphorylation [34] and signaling interactions [35]. To conservatively define the LoF of enhancers, we used deletions from 2,054 whole-genomes to identify enhancers that can be homozygously deleted without obvious fitness defects as LoF-tolerant. We trained a random forest model to learn the characteristic properties of disease-causing potential of enhancers in MegaNet to predict the LoF-tolerance of all enhancers in the human genome. Thus, the LoF-tolerance scores of enhancers provided in this study can significantly facilitate the interpretation and prioritization of non-coding sequence variants for disease and functional studies.

## Results

### Construction of MegaNet

Integration of transcription factor (TF) binding profiles, chromatin features and expression data has revealed the architecture of regulatory networks [36–40]. Availability of tissue-specific annotations has also enabled the construction of tissue-specific regulatory networks. Cao et al. utilized enhancers identified from ENCODE and Roadmap Epigenomics projects [14–16]. They collected ChIP-seq data for H3K4me1, H3K27ac, H3K27me3, DNase-seq together with ChromHMM-predicted active enhancers to generate a union set of enhancers. Using this set of enhancers, they developed a computational model considering the joint effect of the above enhancer features and their correlation to the gene expression to predict the enhancer-target regulation. Importantly, they used ChIA-PET, Hi-C and eQTLs as the gold standard to train their model and connect enhancers with their downstream target genes [41]. In order to systematically evaluate the LoF-tolerance of enhancers in tissue-specific regulatory networks, we collected 246,028 unique enhancers predicted to regulate 19,170 genes from enhancer-target networks [41]. We constructed an integrated mega network (MegaNet) for joint assessment of the enhancer properties in the enhancer-gene regulation networks [41] and gene centrality in the gene-gene interaction networks [13]. The gene-gene interactions in MegaNet consist of protein-protein interactions obtained by high-throughput yeast two hybrid system [32], metabolic interactions obtained by compound-reaction based interactions [33], phosphorylation interactions by direct kinase-substrate interactions [34] and signaling interactions from SignaLink [35].

**Fig 1:**
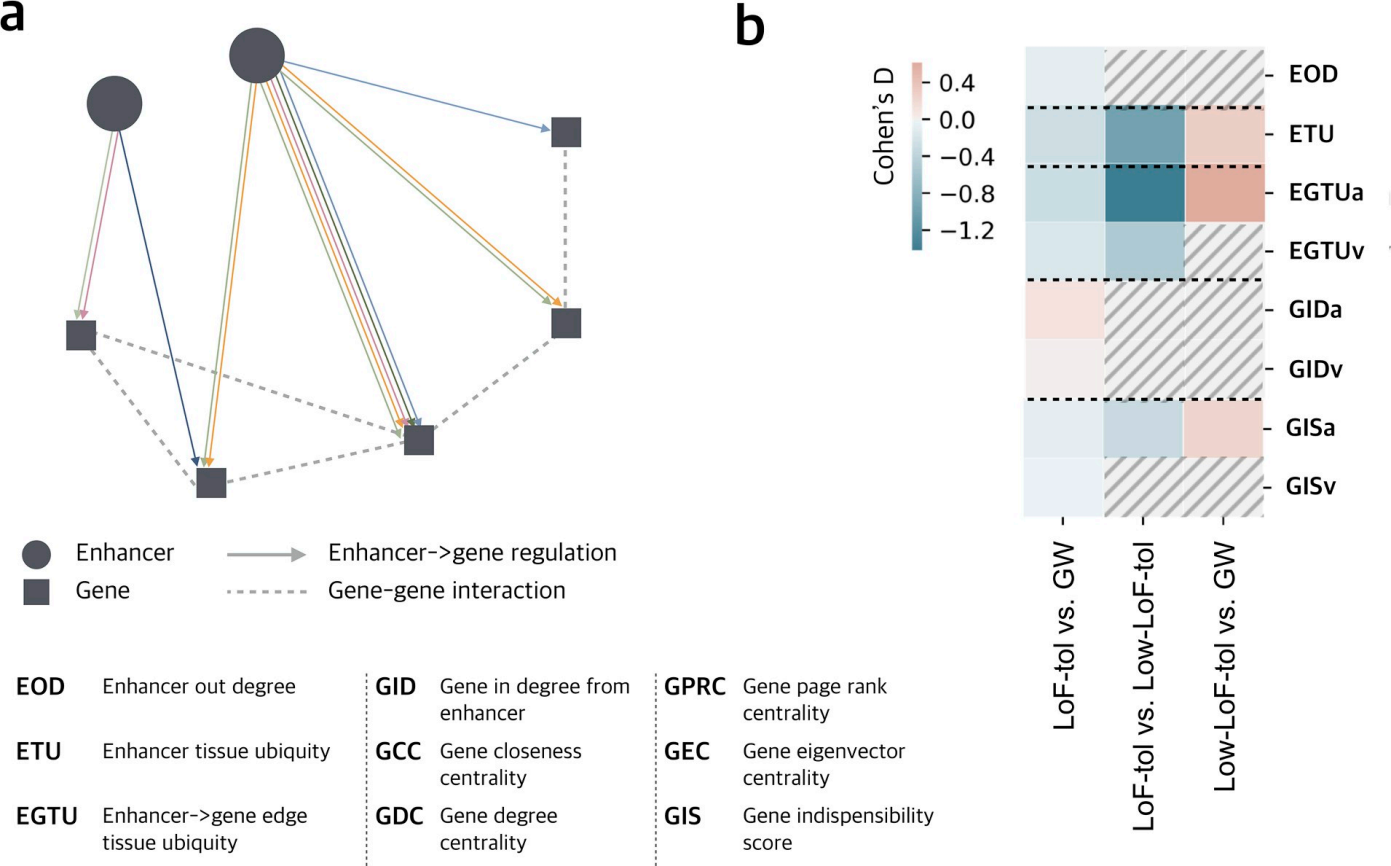
MegaNet features. a) Schema of the MegaNet, circle and square represent nodes for enhancers and genes, respectively, and colored directed arrows are enhancer->gene regulation edges. Different colors represent the interactions active in different tissues. Dashed lines represent the gene-gene interactions. b) Comparison of network features between LoF-tolerant vs. genome-wide, LoF-tolerant vs. Low-LoF-tolerance and Low-LoF-tolerance vs. genome-wide enhancers. Significant comparison (Wilcoxon rank sum test, P-value < 0.05) are shown in color while non-significant ones are marked by dashed lines. Effective sizes of each comparison are shown by Cohen’s D in color scale. Positive Cohen’s D stands for higher average while negative values stand for lower average. LoF-tol, Low-LoF-tol and GW represent LoF-tolerant, low-LoF-tolerance and genome-wide respectively. ‘a’ and ‘v’ stand for the average and variance for the corresponding features in a), detailed feature description is in Table 1.

In the MegaNet, enhancers and genes represent the two kinds of nodes. The directed regulation from enhancers to genes and the undirected interactions between genes are the edges. In order to annotate the tissue-specific properties of nodes and edges in the MegaNet, the enhancer->gene regulation edges are weighted by the number of tissues in which they are active and annotated by tissue types (Fig 1A, **Methods**).

### LoF-tolerant enhancers

We adopted the enhancers annotated by Cao et al. [41] which were collected from the ENCODE and Roadmap Epigenomics projects [14, 16]. Since samples in the 1000 Genomes Project consist of individuals without strong disease phenotypes [3, 42], we define enhancers that can be homozygously deleted in those individuals as LoF-tolerant enhancers. This is similar to the approach used previously for identification of LoF-tolerant genes [1, 2, 5]. More specifically, to identify the LoF-tolerant enhancers, we identified deletions which occur homozygously in at least one individual among the 2,504 from the 1000 Genomes Project [43] and intersected them with enhancers. In order to avoid bias introduced by protein-coding regions, deletions that overlap coding exons were excluded. While deletion of parts of enhancers may also lead to loss of their activity, we used a conservative estimate of LoF-tolerant enhancers by only including those that are completely deleted in a homozygous manner. In line with this, our approach also does not include LoF of enhancers by SNVs due to the difficulties in predicting their functional impact. In total, 886 enhancers are identified as LoF-tolerant. The number of LoF-tolerant enhancers per individual genome ranges from 8 to 78 (S1 Fig).

### Enhancers with low LoF-tolerance

In order to train a model that can predict LoF-tolerance scores for all enhancers, it is useful to have a list of enhancers that are less likely to be tolerant to LoF besides the list of LoF-tolerant ones. Although some disease enhancers have been causally related to fitness defects as discussed in the Introduction, they constitute a small set and most other disease enhancers have been identified to be associated rather than causally linked to diseases. Thus, the known set of causal disease enhancers do not provide a large enough set for model training. Another set of enhancers that has been extensively explored for functional importance is those that exhibit extreme evolutionary conservation and are called ultra-conserved enhancers [44]. Initially, it was reasoned that the extreme conservation might be the result of strong negative selection due to the potential functional importance of these elements [45]. However, besides one early study, which showed that deleting a conserved enhancer causes perinatal death in mice [46], most follow-up studies have shown that ultra-conserved enhancers are not likely to be essential in terms of viability. For example, it was shown that deleting ultra-conserved enhancers is not lethal and upon checking a limited number of phenotypes, their deletion did not show visible abnormalities either [47]. However, further follow-up studies found that even though deletion of ultra-conserved enhancers did not cause perinatal death, mice that survived the deletions did show signs of developmental defects after more comprehensively inspecting for phenotypic changes under different conditions. For example, deleting a conserved and *Shh* regulating enhancer resulted in degenerations of skeletal elements in limb bud [48] and deleting an ultra-conserved limb-developmental associated enhancer led to significantly decreased body size in mouse embryos [49]. Dickel et al showed that single enhancer deletions of three out of the four enhancers regulating the Aristaless-related homeobox (ARX/Arx) gene led to decreased overall growth or brain abnormality in transgenic mice [50]. Thus, our current understanding is that while the loss of ultra-conserved enhancers is not likely to be essential in terms of viability, it is likely to lead to fitness defects, which may be subtle under limited laboratory conditions but are selected against during evolution [50]. Therefore, we compiled 49 low-LoF-tolerance enhancers which exhibit extreme conservation and enhancer activity in mouse embryos and are highly likely to cause fitness defects if deleted [44, 51].

### LoF-tolerance and network properties of enhancers

We analyzed the properties of enhancers in MegaNet using enhancer out-degree (EOD, number of genes that an enhancer targets), enhancer tissue ubiquity (ETU, total number of tissues the enhancer is active in), and enhancer->gene edge tissue ubiquity (EGTU, the number of tissues in which the edges are active) (detailed feature description provided in Table 1). ETU describes the total number of tissues that the enhancer is active in, while EGTU describes the number of tissues that an enhancer->gene regulation edge is active in (Fig 1A). We used integration of multiple biological networks to evaluate the functional essentiality of genes [13]. We assigned the gene indispensability scores generated from that study to genes in our network to integrate the gene indispensability (GIS) in the MegaNet. In order to assess the enhancer-gene interaction landscape in the MegaNet, we also calculated the number of enhancers regulating each gene (Gene In-Degree, GID), and other network centrality metrics as additional gene properties (detailed feature description provided in Table 1). Due to the characteristic architecture of regulatory networks, an enhancer can regulate multiple genes and a gene can be regulated by multiple enhancers as well. Enhancers regulating multiple genes will have multiple values for each gene feature. We consider both the mean and variance to represent their values, and they are represented with an extension “a” (average) or “v” (variance). For example, the enhancer on the left in Fig 1A regulates two genes in three different tissues. The ETU of the enhancer is 3 while the EGTU is a collection of (2,1). The EGTUa for the enhancer will be 1.5 and EGTUv will be 0.25 (**Methods**).

**Table 1:** Summary of network features.

Acronym	Features	Type	Info
EOD	Enhancer out degree	Exact value	Number of genes that an enhancer regulates.
ETU	Enhancer tissue ubiquity	Exact value	Total number of tissues that the enhancer is active in.
EGTUa	Enhancer->gene edge tissue ubiquity	Average	Edges between enhancer and gene are weighted by number of tissues, EGTUa is the average weight of edges for each enhancer.
EGTUv		Variance	
GIDa	Gene in degree from enhancer	Average	Number of enhancers that regulate the gene.
GIDv		Variance	
GCCa	Gene closeness centrality	Average	Closeness centrality of a node u is the reciprocal of the sum of the shortest path distances from u to all n-1 other nodes.
GCCv		Variance	
GDCa	Gene degree centrality	Average	The fraction of nodes that the gene is connected to (including both genes and enhancers).
GDCv		Variance	
GPRCa	Gene page rank centrality	Average	PageRank computes a ranking of the nodes in the graph based on the structure of the incoming links
GPRCv		Variance	
GECa	Gene eigenvector centrality	Average	Eigenvector centrality computes the centrality for a node based on the centrality of its neighbors
GECv		Variance	
GISa	Gene indispensibility score	Average	Khurana et al. 2013a
GISv		Variance	

#### LoF-tolerant enhancers are more tissue-specific and regulate fewer, more dispensable genes

We compared the network properties of enhancers with high vs. low LoF-tolerance and genome-wide expectation (GW, all other enhancers in the MegaNet). We find that LoF-tolerant enhancers regulate significantly fewer genes (i.e., they have lower EOD) compared to genome-wide expectation (Wilcoxon rank sum test P-value = 0.025) and are active in fewer tissues (ETU) compared to both genome-wide expectation and low-LoF-tolerance enhancers (Fig 1B, S3B Fig, Wilcoxon rank sum test P-value = 5.674e-16 and 1.272e-10 respectively). In addition, genes regulated by LoF-tolerant enhancers are more dispensable (lower average gene indispensability score, GISa) compared to genome-wide expectation and low-LoF-tolerance enhancers. In order to account for enhancers with the same average EGTU, but different variance, we also analyzed the variance of EGTU. Both average edge tissue ubiquity (EGTUa) and its variance (EGTUv) are lower for LoF-tolerant enhancers, indicating that their interactions tend to be more tissue-specific (Fig 1B). Overall, these observations indicate that LoF-tolerant enhancers are in general less versatile in the genome and tend to target specific genes in specific tissues.

#### Genes regulated by LoF-tolerant enhancers are regulated by more enhancers

Interestingly, we observe that LoF-tolerant enhancers have higher average gene in-degree, GIDa compared to genome-wide enhancers (Wilcoxon rank sum test P-value = 0.0055), indicating that the genes that LoF-tolerant enhancers regulate are connected to more enhancers (Fig 1B, Table 1). This is consistent with the idea that enhancers can act redundantly in groups and LoF-tolerant enhancers potentially function redundantly to prevent severe phenotypic effects when one or more enhancers are lost [19, 21, 22, 52].

#### LoF-tolerant enhancers are enriched in immune related cells while those with low LoF-tolerance are enriched in kidney and brain/neuronal stem cells

**Fig 2:**
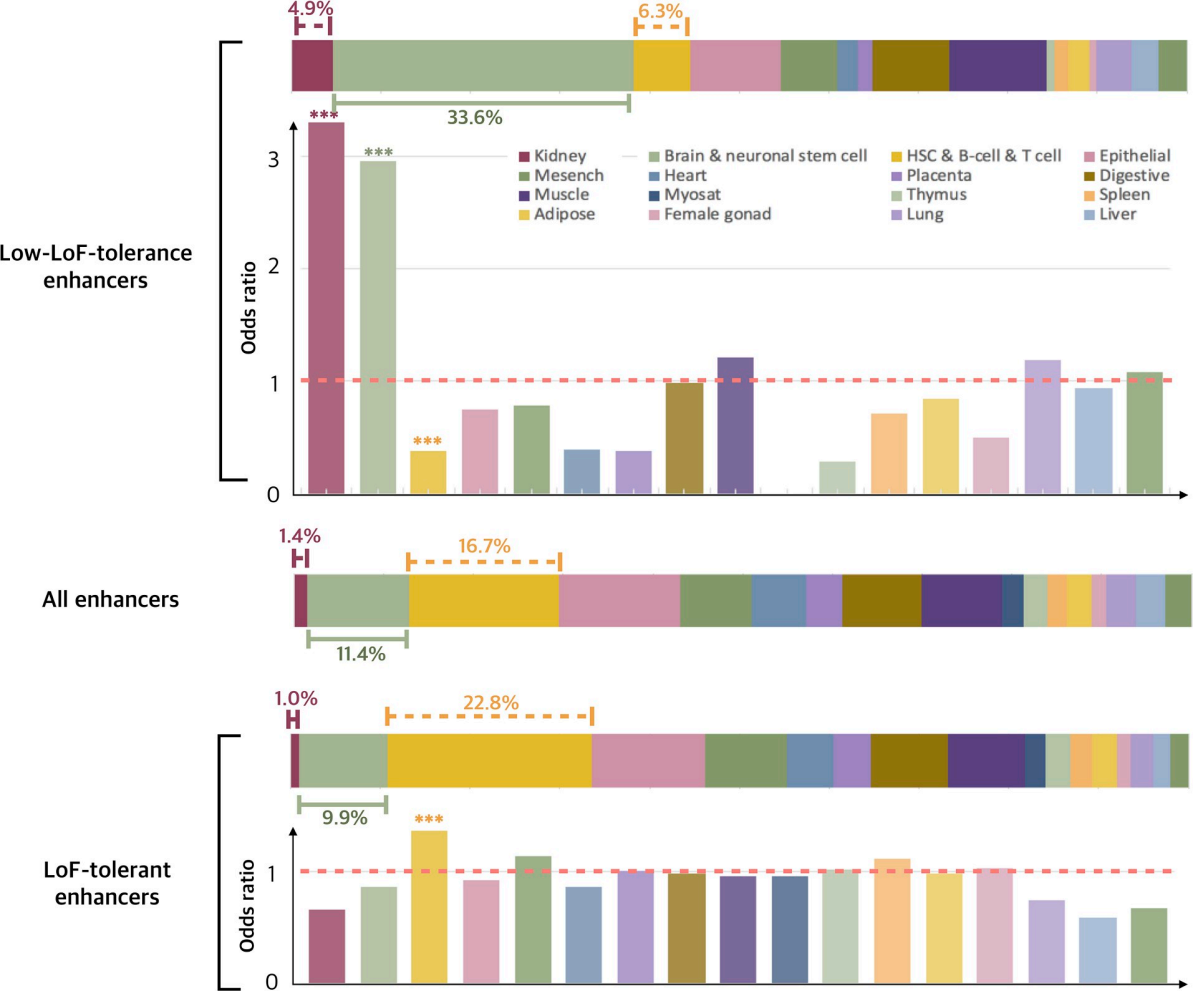
Tissue-specific enhancers. Three horizontal bars separately show the percentage of low-LoF-tolerance, all enhancers and LoF-tolerant enhancers in each tissue type. The matching vertical bar plots show the odds ratios for enrichment of the percentage of low-LoF-tolerance and LoF-tolerant enhancers for each tissue relative to all tissues. (asterisks mark the statistical significance using Fisher’s exact test).

Furthermore, to analyze the tissue-specific properties of enhancers, we extracted the tissue-specific networks from the MegaNet and inspected them individually (S3A Fig). We observe that different tissues exhibit differential enrichment of LoF-tolerant vs. low-LoF-tolerance enhancers. We calculated the odds ratio of LoF-tolerant and low-LoF-tolerance enhancers for each tissue compared to their total numbers across all other tissues respectively (Fig 2). We find that the proportion of enhancers with low LoF-tolerance is significantly enriched in kidney and neuronal stem cell/brain tissues (Fisher’s exact test P-value = 0.010 and 2.80e-11 respectively, Fig 2). Interestingly, this trend is reversed in cells involved in immune response (HSC & B-cell and T-cell), where low-LoF-tolerance enhancers are depleted while LoF-tolerant are enriched (Fisher’s exact test P-value = 4.94e-4 and 1.70e-7, Fig 2).

We also find that genes regulated by LoF-tolerant enhancers are enriched for components of triglyceride-rich plasma lipoprotein particle (adjusted P-value = 3.61e-2 by Benjamini-Hochberg) and inflammasome protein complex (adjusted P-value = 2.22e-2). This is consistent with our observation that LoF-tolerant enhancers are enriched in immune cells. The genes regulated by low-LoF-tolerance enhancers are enriched for processes of embryonic morphogenesis (adjusted P-value = 8.16e-9) and neuron differentiation (adjusted P-value = 1.7e-3), which is consistent with our observation that low-LoF-tolerance enhancers are enriched in brain/neuronal stem cell tissues (S3 Table).

#### TF motifs involved in neurogenesis are enriched in low-LoF-tolerance enhancers

We analyzed the TF binding motifs in LoF-tolerant, GW, and low-LoF-tolerance enhancers for 430 human core motifs (JASPAR2018 [53]). We observe that low-LoF-tolerance enhancers contain more TF motifs, followed by GW, which is followed by LoF-tolerant enhancers (Wilcoxon rank sum test P-values in S8A Fig). This may be related to the higher activity at low-LoF-tolerance enhancers for robustness of the expression of their target genes. We then calculated the enrichment for each motif for LoF-tolerant and low-LoF-tolerance enhancers compared to GW. We observe significant enrichment of 19 motifs in low-LoF-tolerance enhancers (adjusted Fisher exact test P-value < 0.0001). The two TF families with strongest enrichment and lowest p-values are POU domain genes (POU3F1/2/3, POU1F1, POU2F2) and GSX1/2 (S8B Fig). Both of them are involved in neurogenesis [54–56]. Motifs of SOX10, which is critical during embryonic development, are also enriched in low-LoF-tolerance enhancers [57–59]. The enrichment of motifs for neurogenesis-involved TFs is likely related to the enrichment of low-LoF-tolerance enhancers in brain/neuronal stem cell tissues.

### Supervised learning to predict enhancer loss-of-function tolerance

Enhancer->gene regulation occurs in a complex network with interactions between enhancers and genes and among genes. Thus, to systematically predict the LoF tolerance of enhancers, we built a random forest classification model to learn the properties of enhancers and genes in the MegaNet (in total 63 features for 15 tissues as described above and in Table 1, Methods).

**Fig 3:**
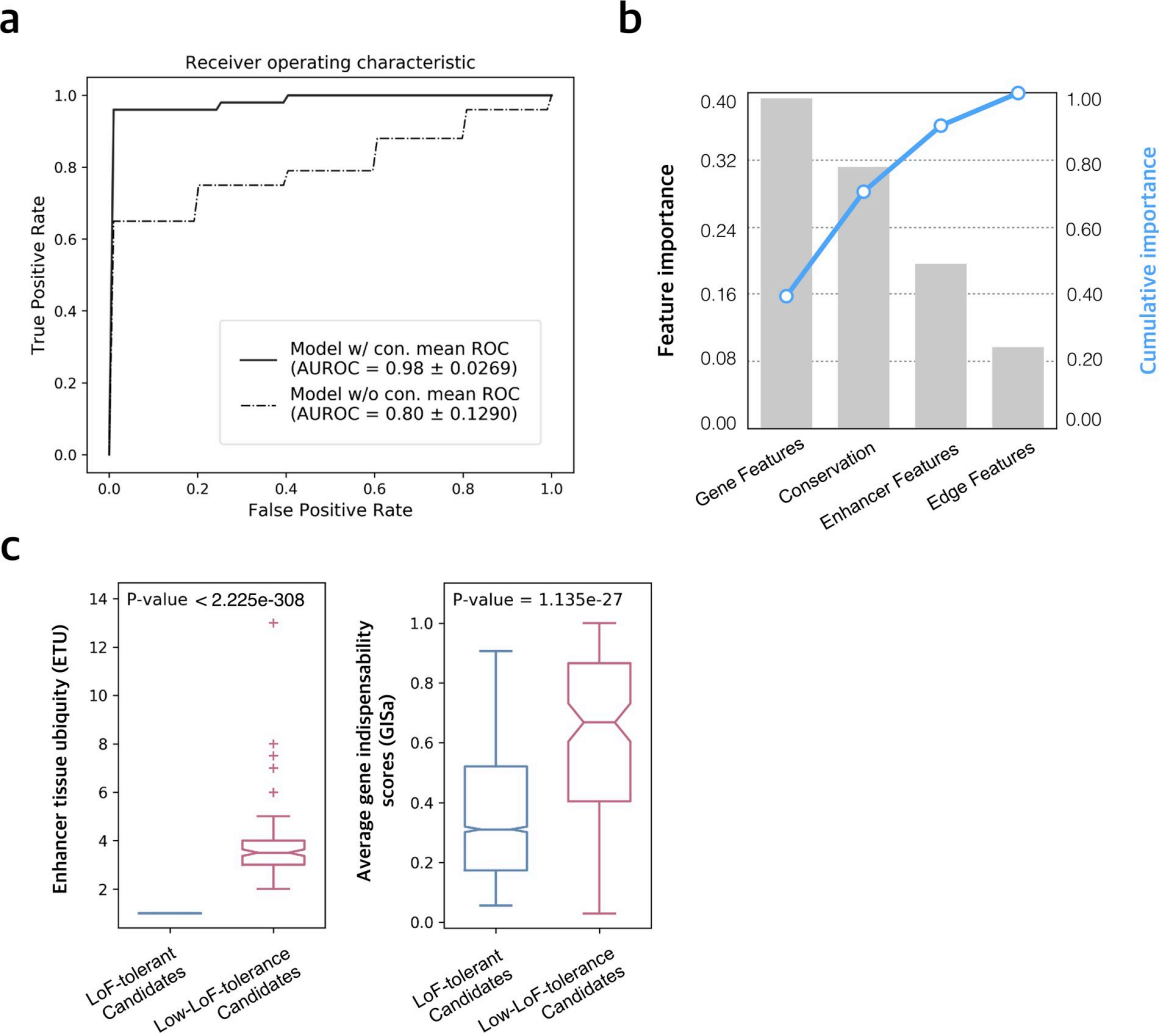
Model performance. a) Stratified 10-fold cross validation mean ROC of the final random forest classification model. Results shown with conservation included and excluded in the feature set. b) Collective feature importance for the classification model. X-axis shows the feature collections. Gene features include gene indispensability scores (GISa/v), and their centrality metrics in the MegaNet (GPRC, GDC, GID, GCC, GEC) and gene in-degrees from each tissue subnetwork. Enhancer features include ETU, EOD in the MegaNet as well as EOD in each tissue subnetwork. Edge features refers to EGTUa/v. See Table 1 for further details of network features. c) Enhancer tissue ubiquity (ETU) and average gene indispensability scores (GISa) for LoF-tolerant and low-LoF-tolerance enhancer candidates.

In order to avoid the prediction bias introduced by unbalanced positive and negative sample sizes, we randomly chose 50 enhancers from the LoF-tolerant enhancer set and used the 49 low-LoF-tolerance enhancers as the negative set to train the model. The process was repeated 50 times to sample all the 886 LoF-tolerant enhancers for training, and the performance of each process was evaluated by stratified 10-fold cross validation (Methods). We thus chose the model from the process which achieved the highest mean area under the receiver operating characteristics (AUROC) as our final model. It achieved an average AUROC of 0.80 +/- 0.129 if evolutionary conservation was not used as a feature and 0.9822 +/- 0.0269 when conservation was also included as a feature. The average AUPRC (area under the precision recall curve) of the final model is 0.9769 +/- 0.0252 (Fig 3A, S4B Fig and Methods). Thus, while inclusion of evolutionary conservation significantly improves the model performance as expected, it performs well even in the absence of this feature. Importantly, a major goal of this study is to decipher the biological differences between enhancers with low vs. high LoF-tolerance as revealed by their network properties, besides the development of the quantitative predictive model for LoF-tolerance scores. Thus, we evaluated the importance of features in the model by mean decrease impurity, which measures the decrease in the weighted impurity of the tree by each feature [60, 61] (Fig 3B and S2 Table). We observe that collectively gene-related features contribute the most to the model (collective importance = 39.4%). Among these features, average gene in-degree of enhancers (GIDa) in neuronal stem cells and average gene indispensability scores (GISa) rank the first. Following the GISa are centrality metrics of genes in the MegaNet such as page rank, degree and closeness centralities. After gene features, evolutionary conservation is next and contributes 31.2%. This is followed by the enhancer properties in MegaNet, including the number of tissues that the enhancers are active in (ETU) and the number of genes they target (EOD), which collectively contribute 19.7%. Finally, the number of tissues that the enhancer-gene regulation edges are active in (EGTU) contribute 9.7%.

### Prediction of novel LoF-tolerant enhancers and validation using PacBio structural variants

We applied our model on all enhancers in the MegaNet, except the ones used in training. Out of 245,093 enhancers tested, 3,519 are predicted to be tolerant to LoF with high LoF-tolerance probability (P_LoF-tol._ > 0.95), while 129 are predicted to be have low tolerance to LoF with very low LoF-tolerance probability (P_LoF-tol._ < 0.05, S2 Table). The predicted low-LoF-tolerance candidates show similar patterns to the ones in the training set as they tend to be active in more tissues (P-value < 2.22e-308) and regulate genes that are more indispensable (P-value = 1.135e-27) compared to LoF-tolerant candidates (Fig 3C, Methods).

**Fig 4:**
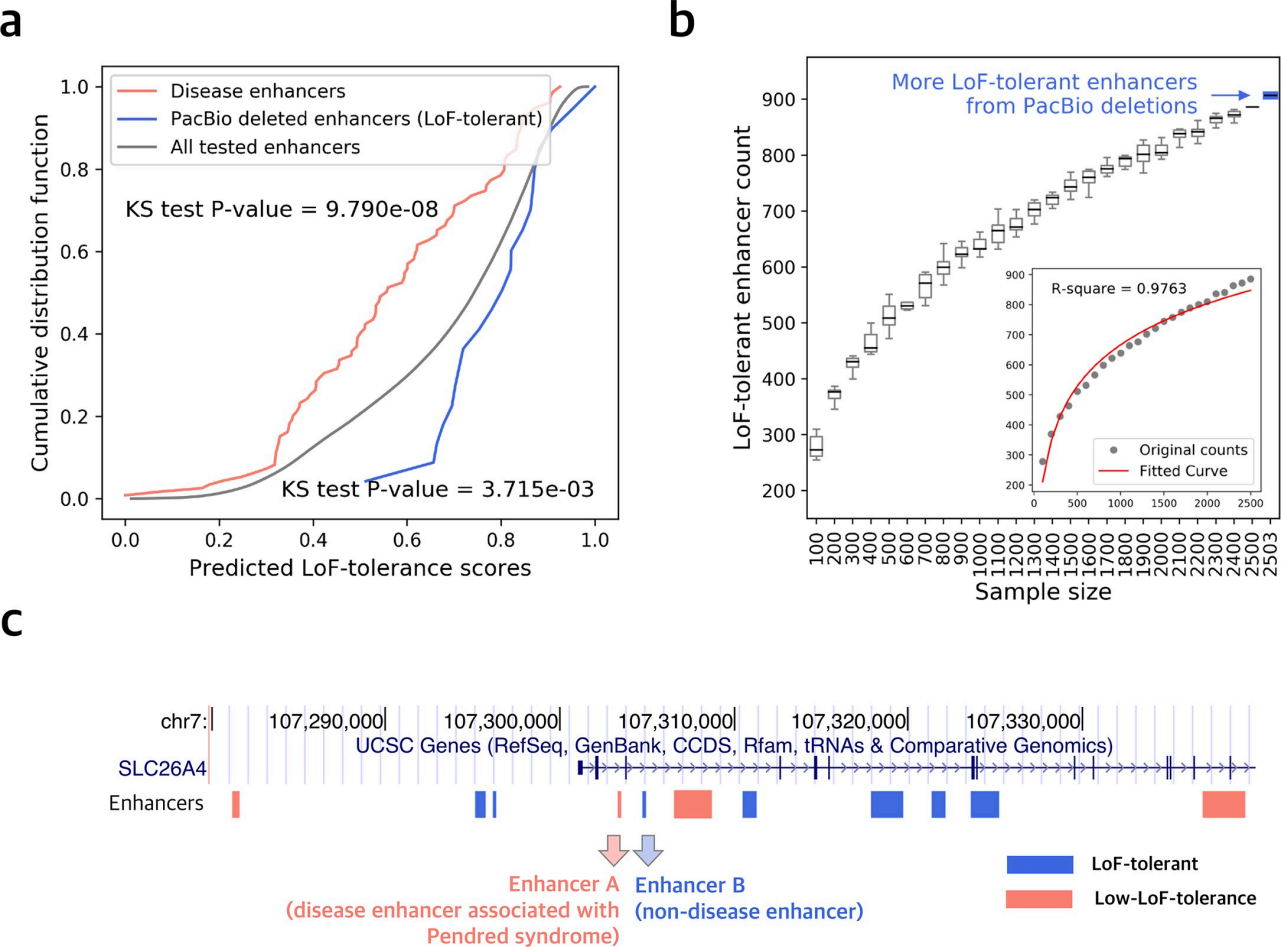
Validation using PacBio SVs and disease enhancers. a) Cumulative distribution function for LoF-tolerant scores for disease enhancers (red), all tested enhancers (grey), PacBio deleted enhancers (blue). KS-test P-values are between disease enhancers vs. all tested and PacBio enhancers vs. all tested. b) Number of observed LoF-tolerant enhancers with increasing sample size. On the x-axis, 2503 includes the LoF-tolerant enhancers observed from 3 additional individuals sequenced using PacBio. c) Genome region of *SLC26A4* and part of the enhancers regulating it. Blue denotes the predicted LoF-tolerant enhancers, while red is for predicted low-LoF-tolerance enhancers.

Overall, in addition to the 886 homozygously deleted LoF-tolerant enhancers used in training, our model predicts additional 3,519 highly confident LoF-tolerant enhancers (P_LoF-tol._ > 0.95). We postulate that many of these enhancers have not yet been detected as LoF-tolerant because of (a) the limited sample size of whole-genome sequences and (b) undetected deletions by short-read sequencing due to the limited mappability of short reads in repetitive and complex regions. In particular, recent studies have pointed out that the map of genomic deletions with Illumina short-reads is highly incomplete. The longer sequencing reads in PacBio technology enabled the detection of many additional SVs (including deletions), particularly in high-repeat regions (24,825 as opposed to 10,884 per human genome) [62–65]. We tested the performance of our method on homozygously deleted enhancers obtained from a combination of PacBio long-reads and Illumina short-reads [65]. We found 21 novel enhancers completely deleted in a homozygous fashion in the three individuals sequenced by Chaisson et al. Our model predicted significantly higher LoF-tolerance probability scores for these enhancers than the genome average (Kolmogorov-Smirnov test P-value = 3.715e-3, Fig 4A). This result shows that the scores predicted by our model can help with identification of LoF-tolerant enhancers even in the absence of large numbers of whole-genomes and incomplete maps of genomic deletions generated using Illumina short-reads.

In order to estimate how many LoF-tolerant enhancers we may expect to obtain as more whole-genomes are sequenced, we randomly chose increasing numbers of genomes in sets of 100 from 2,504 whole-genomes and calculated the number of LoF-tolerant enhancers discovered. Our power calculations using this sub-sampling approach show that the number of LoF-tolerant enhancers is likely to increase exponentially as more genomes are sequenced (Fig 4B). However, sequencing all human genomes to find all the LoF-tolerant enhancers is still infeasible even with short-reads sequencing, let alone more expensive and time-consuming long-reads sequencing. Thus, our model can serve as a practical method to predict which enhancers will be more prone to LoF-tolerance and in the interpretation of disease-associated non-coding variants as discussed below.

### Predicted low-LoF-tolerance enhancers and disease risk

In order to evaluate if our model can predict disease-causing regulatory elements, we inspected some prominent examples of enhancers that have been causally related to severe diseases. Previous studies have shown that a single nucleotide mutation in an enhancer regulating *SLC26A4* can cause decreased enhancer activity leading to repression of gene expression [66], which in turn is associated with Pendred syndrome [67, 68]. Pendred syndrome is a disorder associated with hearing loss caused by abnormalities of inner ear [69, 70]. This enhancer (Enhancer A, Fig 4C) is predicted to have low LoF-tolerance by our model with P_LoF-tol._ = 0.35 (P_LoF-tol_ < 0.5), consistent with its loss of function leading to the disease. In contrast, a neighboring enhancer (Enhancer B), which is 1.2 kbp away is predicted to be LoF-tolerant (P_LoF-tol._ = 0.91). This result shows that our model can differentiate between high vs. low LoF-tolerance of enhancers even when they regulate the same gene.

In another prominent example of enhancers related to severe diseases, *ZIC3* is a protein-coding gene in the ZIC family of C2H2-type zinc finger proteins, acting as a transcriptional activator in the early stages of determining body left-right asymmetry. Mutations in *ZIC3* have been found in X-linked heterotaxy syndrome and isolated congenital heart disease (CHD) [71, 72]. Homozygous mutations in *ZIC3* in mice result in 50% embryonic lethality and live born mice exhibit severe congenital heart defects, pulmonary reversal or isomerism [73]. Out of 33 enhancers that regulate this gene, 18 are predicted to have low LoF-tolerance by our model with average P_LoF-tol._ = 0.31. Previous studies have found 8 LoF mutations in coding regions of *ZIC3* related to the heterotaxy, however, they only explained ~1% of the cases [72]. Therefore, the enhancers predicted to have low LoF-tolerance by our model may provide potential novel susceptibility loci for the study of X-linked heterotaxy and CHD.

These results suggest that the LoF-tolerance probabilities predicted by our model can provide a powerful reference for disease and clinical studies.

To analyze the LoF-tolerance scores for different types of diseases, we extracted a set of disease-associated enhancers from the manually curated DiseaseEnhancer database [74]. This database contains a mixture of enhancers with disease associations and a subset with causal links to disease since the authors looked for multiple evidences, including mechanistic characterization of genetic alterations such as disruption of TF binding [74]. While keeping this limitation in mind, we examined the LoF-tolerance scores predicted by our model for the 90 disease enhancers matched in MegaNet (Methods). We find that these enhancers have significantly lower LoF-tolerance probabilities relative to all the enhancers (Kolmogorov-Smirnov test P-value = 9.790e-8) (Fig 4B). We further categorized these enhancers into different disease groups, for example, obesity, skin diseases, neurological disorders, artery diseases, immune disorders, and developmental diseases. We find that skin disease related enhancers have higher LoF-tolerance probability scores (Wilcoxon rank sum test P-value = 0.024, S5A Fig), while psychological disorders related enhancers have lower LoF-tolerance scores (Wilcoxon rank sum test P-value = 0.019, S5A Fig).

### Non-conserved enhancers may exhibit low tolerance to LoF

We find that the LoF-tolerance and PhastCon scores are negatively correlated (Spearman correlation coefficient = -0.33, P-value < 2.22e-308) as expected since conservation is the second important feature for LoF-tolerance prediction. However, they are still different metrics and enhancers with low conservation can still have low LoF-tolerance. From the disease enhancer set described in the previous section, there are 12/39 enhancers with conservation < 0.065 (median of all enhancer PhastCon scores) [75] yet they are predicted to have low LoF-tolerance by our model. One example is an enhancer regulating the gene *SOX10*. An SNV (rs533778281) in this enhancer has been shown to decrease the enhancer activity by disrupting SOX10 binding, which in turn leads to Hirschsprung disease [59]. Hirschsprung disease is a birth defect in which nerves in the intestine are not developed normally causing difficulty in intestine movement. The enhancer reported in the study covers two enhancers in our dataset with P_LoF-tol_ = 0.33 and 0.27, hence they are predicted to be low-LoF-tolerance enhancer candidates (S5B Fig), even though the conservation for this enhancer region is low (PhastCon score = 0.024 and 0.062 respectively). The features related to the low LoF-tolerance of these enhancers in our model include high gene indispensability scores (GISa = 0.62 and 0.61 respectively) and regulation of multiple genes in the MegaNet (EOD = 12 and 10 respectively). This example further shows that our model can help prioritize and interpret disease variants using gene and MegaNet features beyond evolutionary conservation alone.

## Discussion

In this study, we constructed a unified human regulatory network (MegaNet) by integrating tissue-specific enhancer-target networks and gene-gene interactions. To define enhancers that may be tolerant to LoF in the genome, we used deletions from the 1000 Genomes Project. We describe the differences between LoF-tolerant and enhancers with low LoF-tolerance in the MegaNet. We observe that LoF-tolerant enhancers regulate fewer genes and tend to be more tissue-specific. We also find that the genes regulated by LoF-tolerant enhancers tend to be regulated by more enhancers, indicating enhancer redundancy in the network. We developed a supervised learning method to predict the LoF-tolerance of all enhancers in the human genome. Independent data sets obtained using long-read sequences and known sets of disease enhancers provide validation for the LoF-tolerance scores predicted by our model.

GWAS have revealed that the majority of the variants associated with complex diseases reside in non-coding regions of the genome [27, 28, 76]. Moreover, even though whole-exome sequencing has revealed causal variants for many Mendelian disorders [77], the genes underlying ~50% of Mendelian phenotypes are still unknown [78]. It is possible that regions excluded from exome sequencing, namely non-coding regions, harbor the variants explaining many of the remaining unexplained cases [79]. Major international efforts such as the UK Biobank and TOPMed (NHLBI Trans-Omics for Precision Medicine) aim to use whole-genome sequencing to uncover disease variants [4, 80–84]. The LoF-tolerance scores for enhancers provided here can significantly facilitate the interpretation and prioritization of non-coding sequence variants in whole-genome sequencing studies.

We note that the LoF-tolerance scores provided here predict how well the loss of enhancer would be tolerated by the organism while other scoring schemes (such as FunSeq2 [85, 86], FUN-LDA[87], CADD [88, 89], DeepSea [90], etc.) aim to predict the functional impact of mutations on enhancer activity, e.g. binding of TFs and downstream gene expression, and if that would be associated with fitness defects. As an example, a mutation may lead to loss of activity giving it high functional impact score but that might not lead to fitness defects. We calculated the variance of FunSeq2 scores of mutations in each enhancer (S7A Fig). The FunSeq2 score varies within each enhancer for the same predicted LoF-tolerance scores. We also show this using a specific enhancer as example (S7B Fig). Thus, for researchers investigating the function of non-coding variants in enhancers, methods like FUN-LDA and FunSeq2 can be used to find the functional effect scores of their mutations, while our scoring scheme can be used to further check how well losing the enhancer will be tolerated by individuals. Another important difference of our approach is that LoF-tolerance scores can be used to predict the consequences of structural variants (such as deletions) that are likely to disrupt enhancers by predicting how well the loss of enhancer would be tolerated.

## Materials and methods

### Constructing MegaNet

Enhancer-gene networks in different tissues were obtained from the ENCODE+Roadmap LASSO dataset in Cao et al. [41] (http://yiplab.cse.cuhk.edu.hk/jeme/). In Cao et al, they collected ChIP-seq data for H3k4me1, H3K27ac, H3K27me3, DNase-seq together with ChromHMM-predicted active enhancers to generate a union set of enhancers. We grouped 127 Roadmap tissue types by the given sample group into 19 tissue groups and discarded ungrouped cell types (S1 Table).

To construct the MegaNet, we first added all directed enhancer->gene edges without replica into the network, then weighted the enhancer->gene by the number of tissues in which they are active and annotated by tissue types. Then we added gene-gene interactions, since gene interaction edges are undirected, we added such edges by adding them twice in the opposite direction in the network. In such a way, the degree centralities of genes are not affected (it counts the number of neighboring nodes instead of the number of edges), also the closeness/page rank/eigenvector centralities will be properly calculated.

### Curation of LoF-tolerant and low-LoF-tolerance enhancers

In order to identify LoF-tolerant enhancers, we first identified all deletions existing in a homozygous state in any one individual in the 1000 Genomes Phase 3 data [43]. We excluded any deletion overlapping coding exon regions and then intersected the remaining deletions with enhancer coordinates to obtain our list of 886 LoF-tolerant enhancers. Only enhancers that are 100% deleted were included.

In order to identify low-LoF-tolerance enhancers, we started with ultra-conserved elements and retained only those showing consistent reporter gene expression [44, 50, 51, 91]. We intersected the remaining elements with enhancer coordinates in our dataset, keeping only those with >50% reciprocal overlap. In total, we define 49 low-LoF-tolerance enhancers.

We compared the length distributions of enhancers and deletions (S6 Fig). The average length of deletions is much longer than enhancers. Thus, LoF-tolerant enhancers are likely not biased towards shorter enhancers (shorter enhancers are more likely to be completely deleted). To be more stringent, we still excluded the length of enhancers as a feature in the following analysis.

#### Transcription factor binding site analysis

We extracted homo sapiens core 430 TFs from JASPAR2018. We used matchMotifs from motifmatchr [92] with default settings. The percentage of TF binding sites present was calculated by the number of enhancers containing the motif divided by the total number of enhancers in each category.

### Tissue-specific subnetworks

To distinguish enhancer activity differences between tissues, we extracted tissue-specific networks from the MegaNet. Enhancers in HSC & B-cell and Epithelial tissues exhibit significant differences in tissue-specific network properties between LoF-tolerant and low-LoF-tolerance enhancers (Wilcoxon rank sum test P-value < 0.05, S3C Fig).

### Collecting features for the model

Besides the tissue specificity information of enhancers, we also used the gene centralities and gene indispensability scores [13] as measurements for gene priority in the network. In order to only consider the direct interactions between gene pairs, indirect interactions, genetic interaction and regulatory interactions, were excluded from our integrated network. Enhancer-target network features were calculated using Python networkX package [93]. Conservation scores for sequence were obtained from PhastCons [75].

Detailed information about network features is provided in Table 1. For enhancers that regulate multiple genes, to transform gene features for those regulated genes into an enhancer feature, we took both the average and variance for each gene features and represented it with extension “a” (average) or “v” (variance). For each enhancer, we denote ETU as n, then EGTU is a list of (*e*_1_, *e*_2_, …, *e_n_*). The EGTUa will be $\frac{\sum_{i=1}^{n}e_{i}}{n}$, and the EGTUv is $\frac{\sum_{i=1}^{n}{\left(e_{i}-EGTUa\right)}^{2}}{n}$.

### Feature selection

To avoid overfitting introduced by features correlated with each other, we calculated the Spearman distance between each feature. We noticed that features for tissue type adipose/epithelial and digestive are strongly correlated with each other, thus only one of them (adipose) was kept for further model building. In addition, tissue type myosat and mesench are mixed with other tissue clusters, so we eliminated them from the final tissue set. In the end, there are in total 15 tissue types considered and 62 features overall.

### Model building and testing

The model was built using tools from Python Scikit-learn package [61]. For each process, we randomly selected 50 LoF-tolerant together with the 49 low-LoF-tolerance, then used random and grid searches to find the best parameters for the random forest classifier. At last, stratified 10-fold cross validation was performed to evaluate the performance of the model in each process. To avoid overfitting, we repeatedly the above processes across all LoF-tolerant enhancers 50 times. Each process generated a mean AUROC, to get an average performance of all the models, the average of the 50 mean AUROCs is 0.9528 +/- 0.0004. Then we chose the model which achieved the highest mean AUROC as our final model. The mean AUROC for this model is 0.9822 +/- 0.0269 (Fig 3A, S4 Fig). Due to the small sample size of low-LoF-tolerance enhancers, we also randomly chose 50 enhancers from neither the LoF-tolerant nor low-LoF-tolerance set as “low-LoF-tolerance” to test overfitting of the model. We performed the same parameter searching and cross validation repeatedly 50 times and obtained average mean AUROCs of 0.5750 +/- 0.0056, indicating that the small sample size for low-LoF-tolerance enhancers did not lead to overfitting.

We applied the model on all other enhancers in the network and predicted their probability to be LoF-tolerant as their LoF-tolerance scores. The predicted LoF-tolerant probabilities are the mean predicted class probabilities of the trees in the forest [61]. Among 245,093 enhancers tested, 186,333 (P_LoF-tol_ > = 0.5) are predicted to be LoF-tolerant enhancers, while 58,760 are predicted to have low LoF-tolerance (P_LoF-tol_ < 0.5).

### Validation

To further validate our observation that there are additional LoF-tolerant enhancers in human genomes, we obtained novel deletions to identify LoF-tolerant enhancers. Those novel deletions were from the 1000 Genomes structural variation consortium where they used integrated structural variation calling methods including both Illumina short reads and PacBio long reads sequencing for three individuals from 1000 Genomes trio studies [65]. In total, we used 12,939 deletions from the PacBio structural variants set that were present in the three children (HG00514, HG00733 and NA19240) from the trio family and intersected them with 1000 Genomes Phase 3 deletions. There are 11,118 novel deletions with less than 80% overlap with the 1000 Genomes Phase 3 deletions. Out of those novel deletions, 21 of them can delete enhancers completely from our enhancer set.

### Disease enhancers

Disease enhancers were collected from Zhang et al. (Zhang et al. 2018). We intersected our enhancers with the 1,059 disease enhancers which defined in Zhang et al., if no overlap found then take the closest neighbored enhancer. After this, keep only the disease enhancers that its target gene from the DiseaseEnhancer matches the enhancer-gene regulation from our dataset. To further filter out the disease enhancers related to somatic variants, we excluded enhancers associated with cancer. In the end, we collected 90 enhancers in our dataset with disease associations.

## Supporting information

S1 Fig Number of LoF-tolerant enhancers per individual from 2,504 genomes. Each individual has on average 28 enhancers (red vertical line) completely and homozygously deleted in the genome.
(TIF)

S2 Fig Comparison of enrichment of rare variants and all polymorphisms between LoF-tolerant and low-LoF-tolerance enhancers and all other enhancers (genome-wide, GW). Upper P-value is for LoF-tolerant vs. GW, while lower P-value is for low-LoF-tolerance vs. GW. The P-values were calculated by Kolmogorov-Smirnov test (KS test).
(TIF)

S3 Fig Network features in the MegaNet and in tissues-specific networks. a) Example sub-networks centered around *S100P* from six tissues. Nodes and edges that are directly connected to *S100P* are shown, LoF-tolerant enhancers are marked in blue circles. *S100P* is involved in gastric cancer network [94, 95] and innate immune system pathways [96, 97]. b) Network features in the MegaNet, significant comparisons are marked by asterisks. c) Each column represents a tissue-specific network comparison between LoF-tolerant vs. low-LoF-tolerance enhancers.
(TIF)

S4 Fig Performance of the final model. a) Stratified 10-fold cross validation mean ROC of the final random forest classification model. Results shown with conservation included and excluded in the feature set. The “Random sampling” line in the figure is the performance for null model using the final model dataset where we take the 50 LoF-tolerant enhancers and randomly chose 50 enhancers from neither the LoF-tolerant nor low-LoF-tolerance set as “low-LoF-tolerance” to test overfitting of the model; b) Precision-recall curve of the final model.
(TIF)

S5 Fig Disease enhancers. a) Predicted LoF-tolerance scores for disease enhancers by disease types. Y-axis is the cumulated percentage of enhancers for the corresponding LoF-tolerance scores on x-axis. Disease types are colored as shown, significant ones (Wilcoxon rank sum test P-value < 0.05) are marked by asterisks. b) Genome region of *SOX10* and part of the enhancers regulating it. Blue denotes the predicted LoF-tolerant enhancers, while red is for predicted low-LoF-tolerance enhancers. PhastCon scores of predicted enhancers are shown in green, annotated as “Vertebrate Cons.”.
(TIF)

S6 Fig Properties of deletions and enhancers. a) Length distribution of homozygous deletions that do not overlap with exons, blue marks the deletions deleting enhancers; b) Length distribution of deleted enhancers (LoF-tolerant enhancers) and all enhancers; c) Density of allele frequency of enhancer-deleting deletions by super populations (LoF-tolerant enhancers). The frequency distributions are significantly different for pair-wise comparisons of the super populations (KS, Kolmogorov–Smirnov test P-value < 0.05) except for comparison between European and South Asian. Allele frequency of LoF-tolerant enhancers are significantly higher in African population which is consistent with allele frequency distribution of all deletions in human genomes; d) Density of allele frequency of all deletions and LoF-tolerant enhancer-deleting deletions among all 1000 Genomes samples. Allele frequencies of LoF-tolerant enhancer-deleting deletions are significantly higher than all, indicating that they are more common in the population (KS test P-value = 8.33e-254).
(TIF)

S7 Fig Variation of FunSeq2 scores: a) Variance of FunSeq2 scores for single nucleotide variants in each enhancer with its predicted LoF-tolerance score. Orange circle indicates the enhancer chosen for exhibition in sub-figure b; b) The genomic location of the example enhancer (chr4:185,585,400–185,586,600) with FunSeq2 scores and conservation accordingly. The example enhancer locates within an intron of *CCDC111* gene and was predicted to be a LoF-tolerant enhancer with a LoF-tolerance score of 0.82. The FunSeq2 scores for mutations in this enhancer range from 0.011 (low functional impact) to 3.34 (high functional impact). The high LoF-tolerance score shows that even if a high functional impact mutation disrupts this enhancer, it will likely be well tolerated and not lead to major fitness defects. c) We found weak negative correlation between eQTL density and our predicted LoF-tolerance scores (SCC = -0.13, P-value < 2.22e-308). This is consistent with our understanding that high density of eQTLs points towards functional importance which corresponds to low LoF-tolerance scores.
(TIF)

S8 Fig Enrichment of TF binding motifs in LoF-tolerant and low-LoF-tolerance enhancers. a) The y-axis shows the number of motifs found in each enhancer. The significant comparisons are marked by asterisks (Wilcoxon rank sum test P-value = 7.14e-11, 1.32e-15 and 9.22e-19 for LoF-tolerant vs. GW, low-LoF-tolerance vs. GW and LoF-tolerant vs. low-LoF-tolerance respectively); b) X-axis shows the motif presence percentage difference between LoF-tolerant vs. GW and low-LoF-tolerance vs. GW. The top 10 significantly enriched TFs in low-LoF-tolerance enhancers (adjusted Fisher exact test P-value < 0.0001) are labeled in red.
(TIF)

S1 Table Categories of ENDODE and Roadmap tissues. (XLSX)

S2 Table Predicted LoF-tolerance scores for all enhancers in this study, and feature importance of the model. (XLSX)

S3 Table Genes regulated by LoF-tolerant and low-LoF-tolerance enhancers. (XLSX)
