# Supplementary figures and images for: Loss-of-function tolerance of enhancers in the human genome
Source: PLoS Genet. 2020 Apr 3;16(4):e1008663. doi: 10.1371/journal.pgen.1008663 (PMC7159235; doi:10.1371/journal.pgen.1008663)

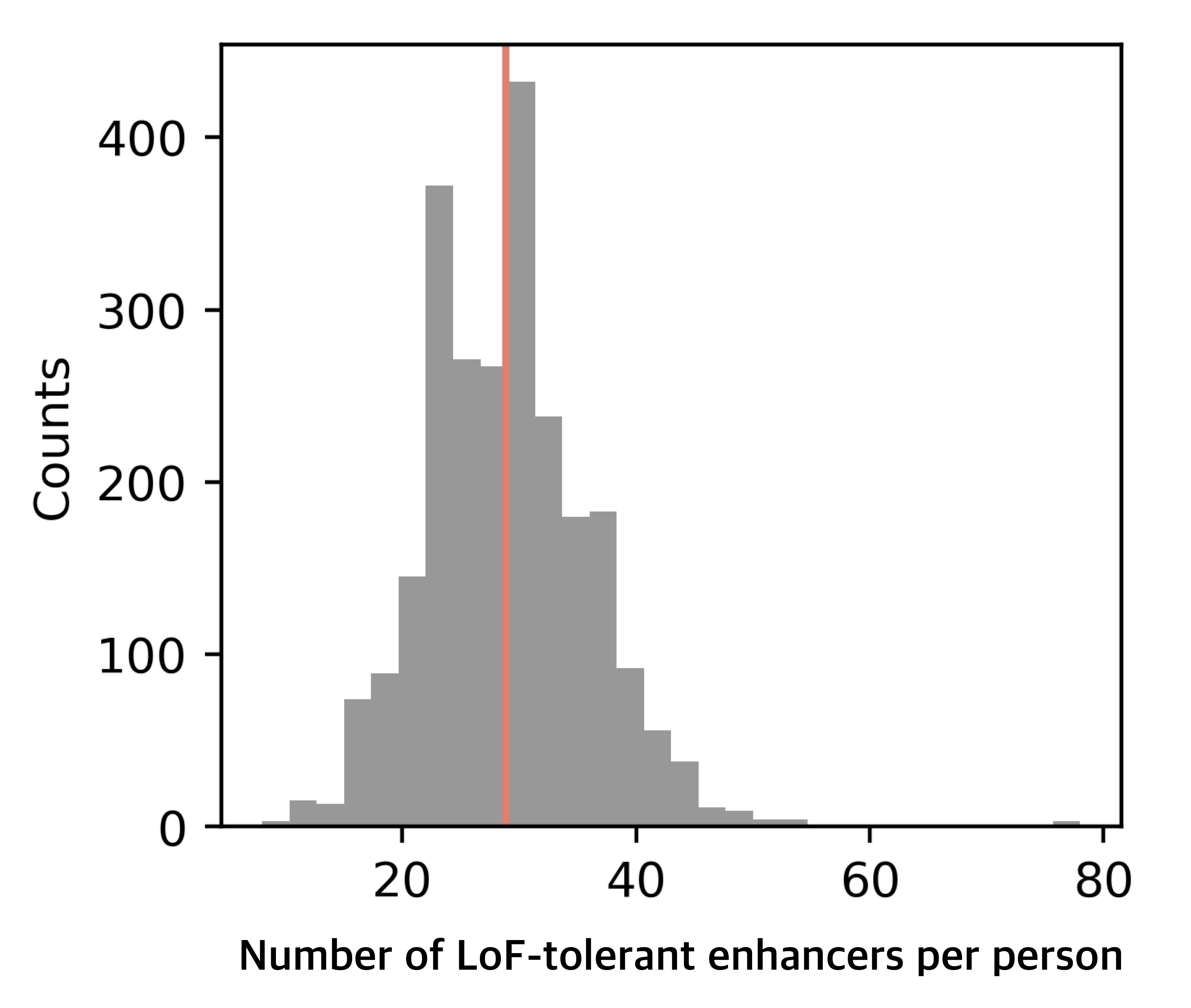

Supplement: S1 Fig — Each individual has on average 28 enhancers (red vertical line) completely and homozygously deleted in the genome. (TIF) [file pgen.1008663.s001.tif]

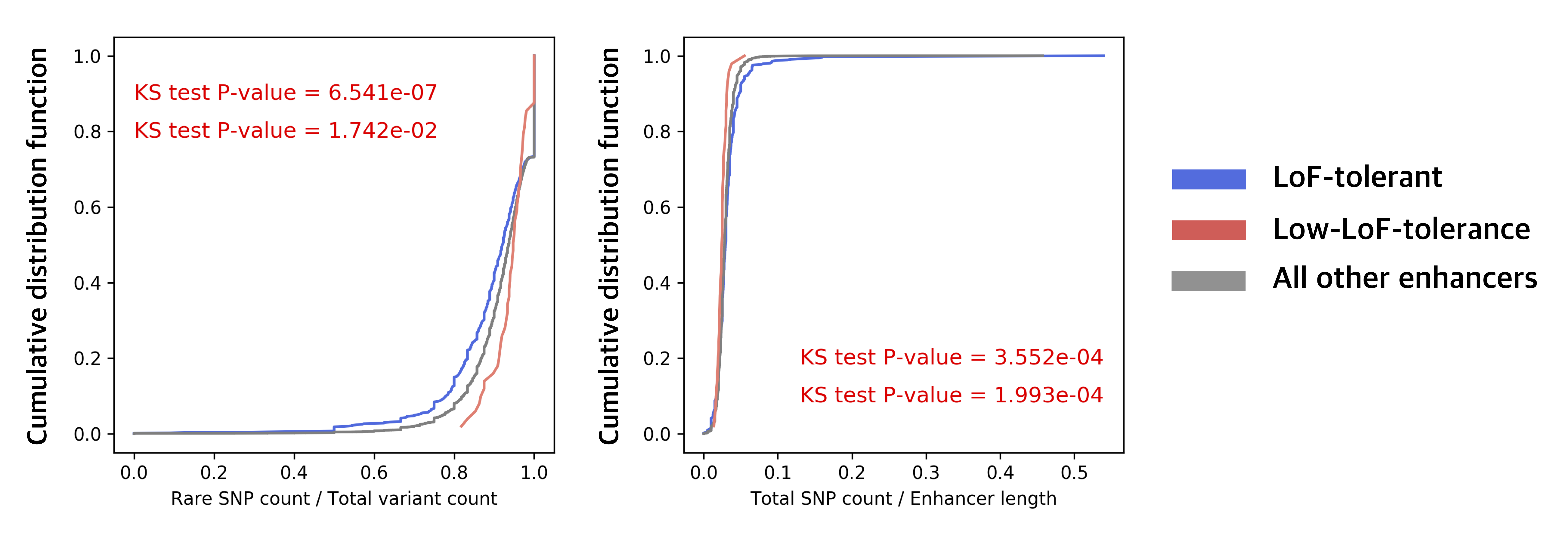

Supplement: S2 Fig — Upper P-value is for LoF-tolerant vs. GW, while lower P-value is for low-LoF-tolerance vs. GW. The P-values were calculated by Kolmogorov-Smirnov test (KS test). (TIF) [file pgen.1008663.s002.tif]

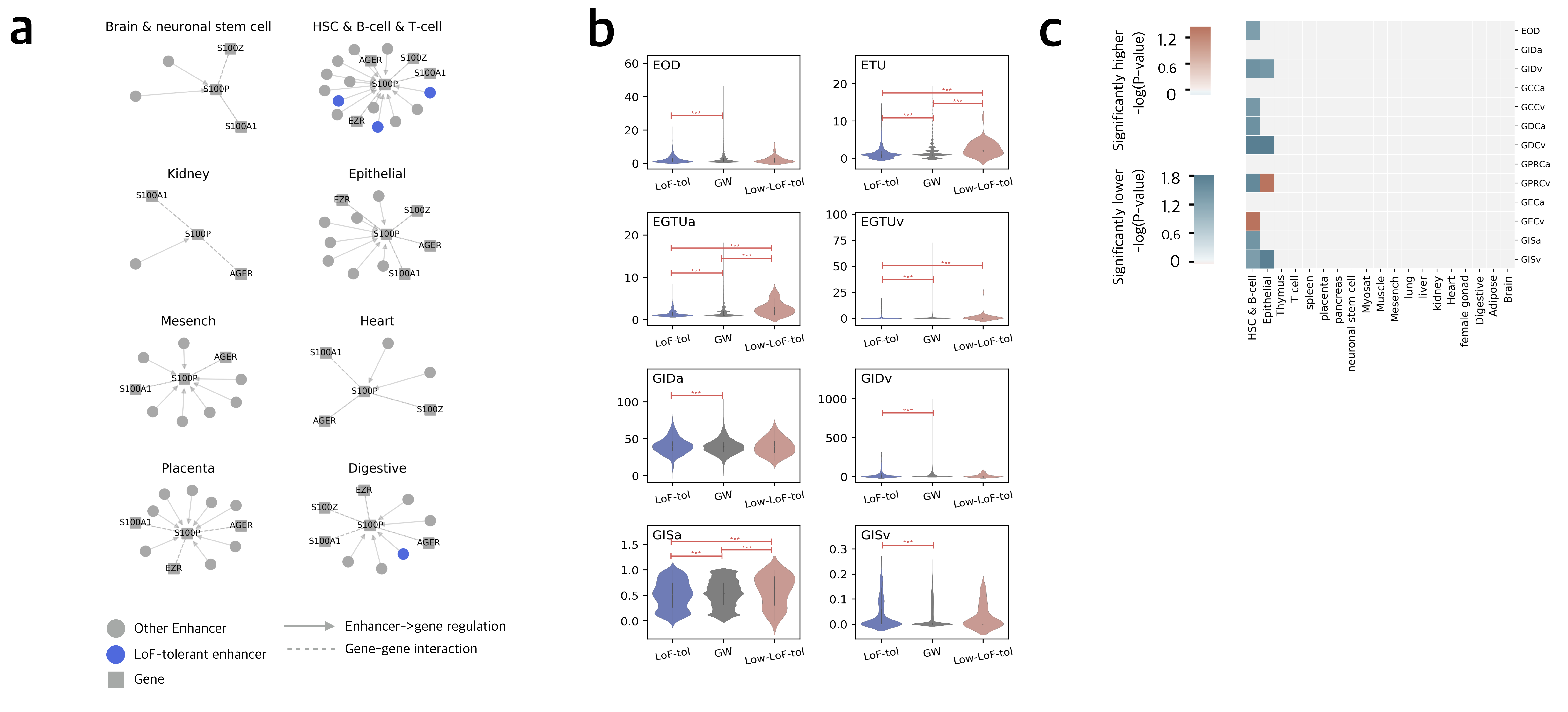

Supplement: S3 Fig — a) Example sub-networks centered around S100P from six tissues. Nodes and edges that are directly connected to S100P are shown, LoF-tolerant enhancers are marked in blue circles. S100P is involved in gastric cancer network [94, 95] and innate immune system pathways [96, 97]. b) Network features in the MegaNet, significant comparisons are marked by asterisks. c) Each column represents a tissue-specific network comparison between LoF-tolerant vs. low-LoF-tolerance enhancers. (TIF) [file pgen.1008663.s003.tif]

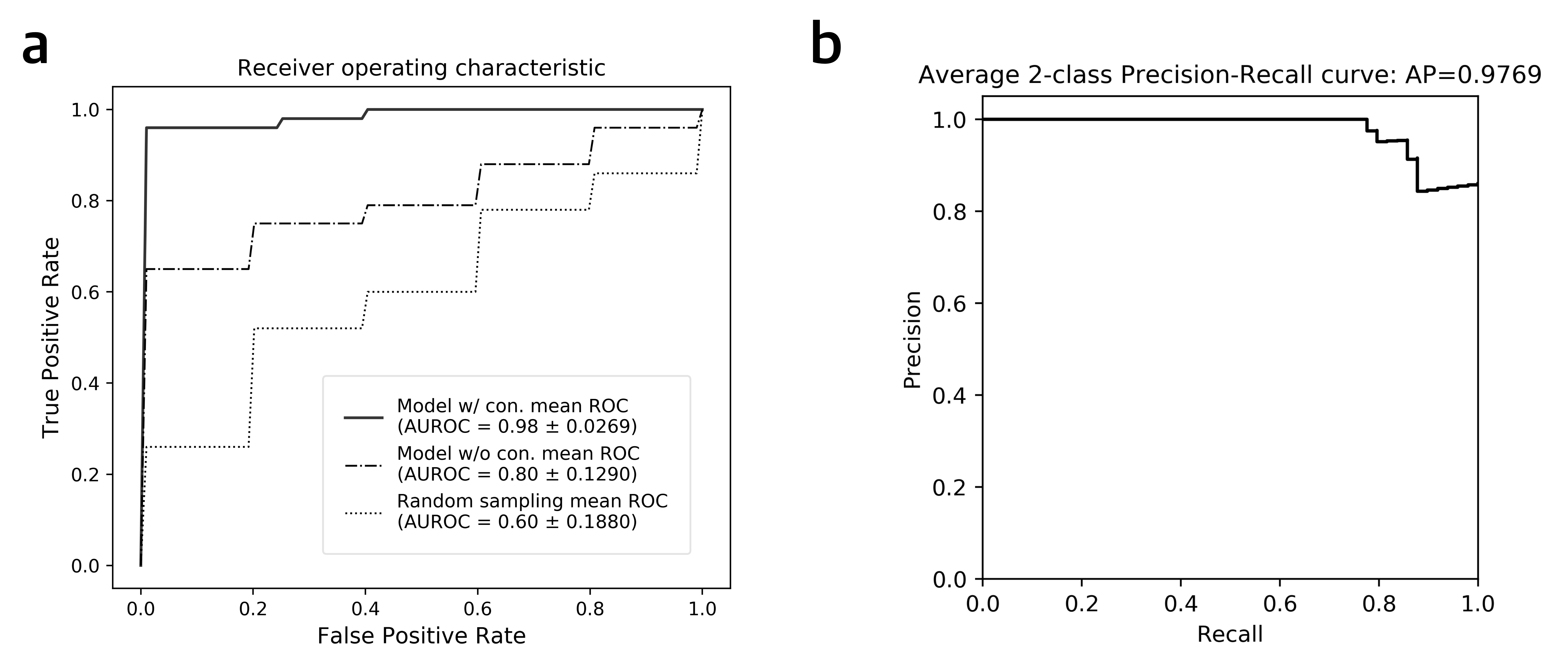

Supplement: S4 Fig — a) Stratified 10-fold cross validation mean ROC of the final random forest classification model. Results shown with conservation included and excluded in the feature set. The “Random sampling” line in the figure is the performance for null model using the final model dataset where we take the 50 LoF-tolerant enhancers and randomly chose 50 enhancers from neither the LoF-tolerant nor low-LoF-tolerance set as “low-LoF-tolerance” to test overfitting of the model; b) Precision-recall curve of the final model. (TIF) [file pgen.1008663.s004.tif]

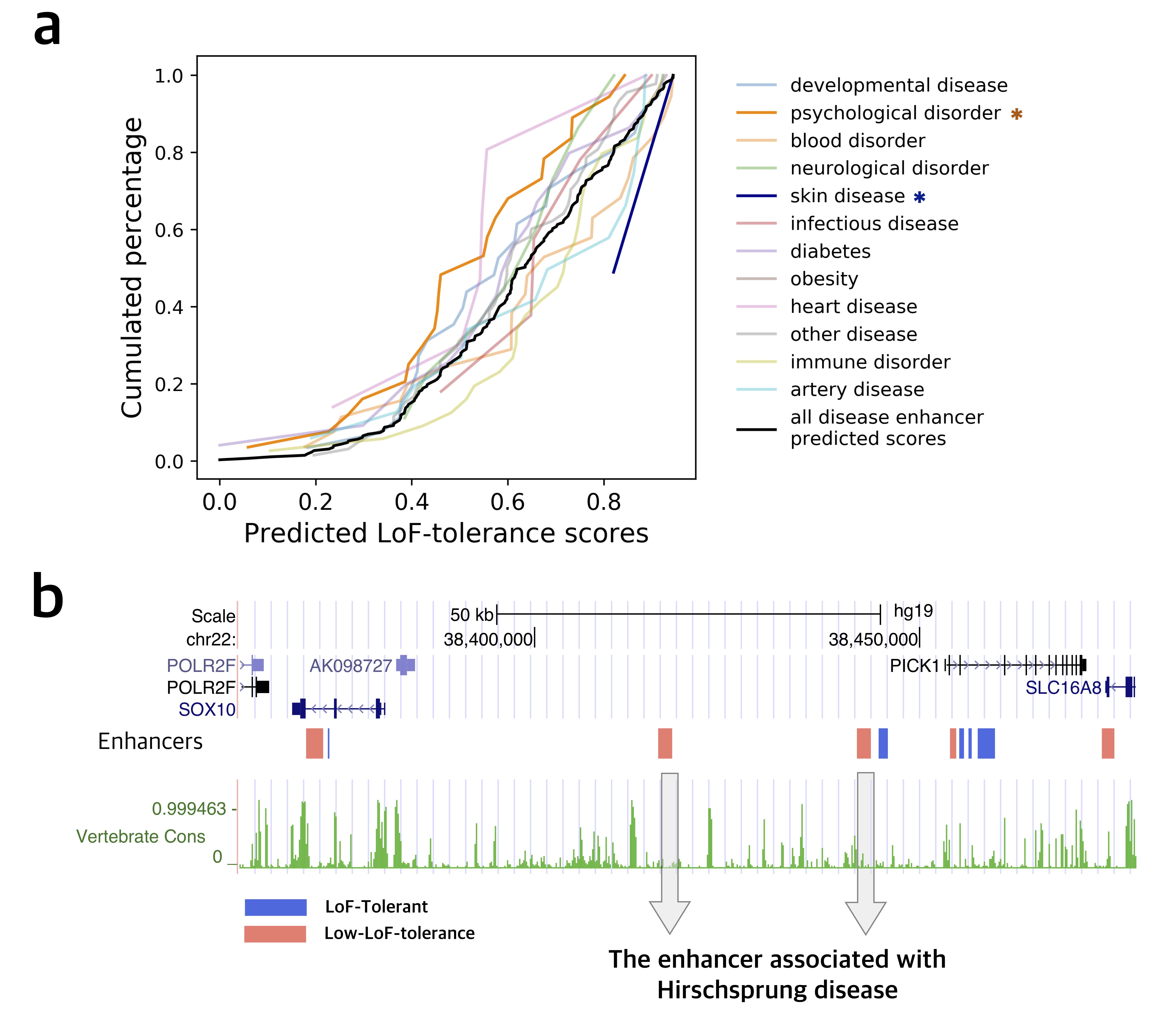

Supplement: S5 Fig — a) Predicted LoF-tolerance scores for disease enhancers by disease types. Y-axis is the cumulated percentage of enhancers for the corresponding LoF-tolerance scores on x-axis. Disease types are colored as shown, significant ones (Wilcoxon rank sum test P-value < 0.05) are marked by asterisks. b) Genome region of SOX10 and part of the enhancers regulating it. Blue denotes the predicted LoF-tolerant enhancers, while red is for predicted low-LoF-tolerance enhancers. PhastCon scores of predicted enhancers are shown in green, annotated as “Vertebrate Cons.”. (TIF) [file pgen.1008663.s005.tif]

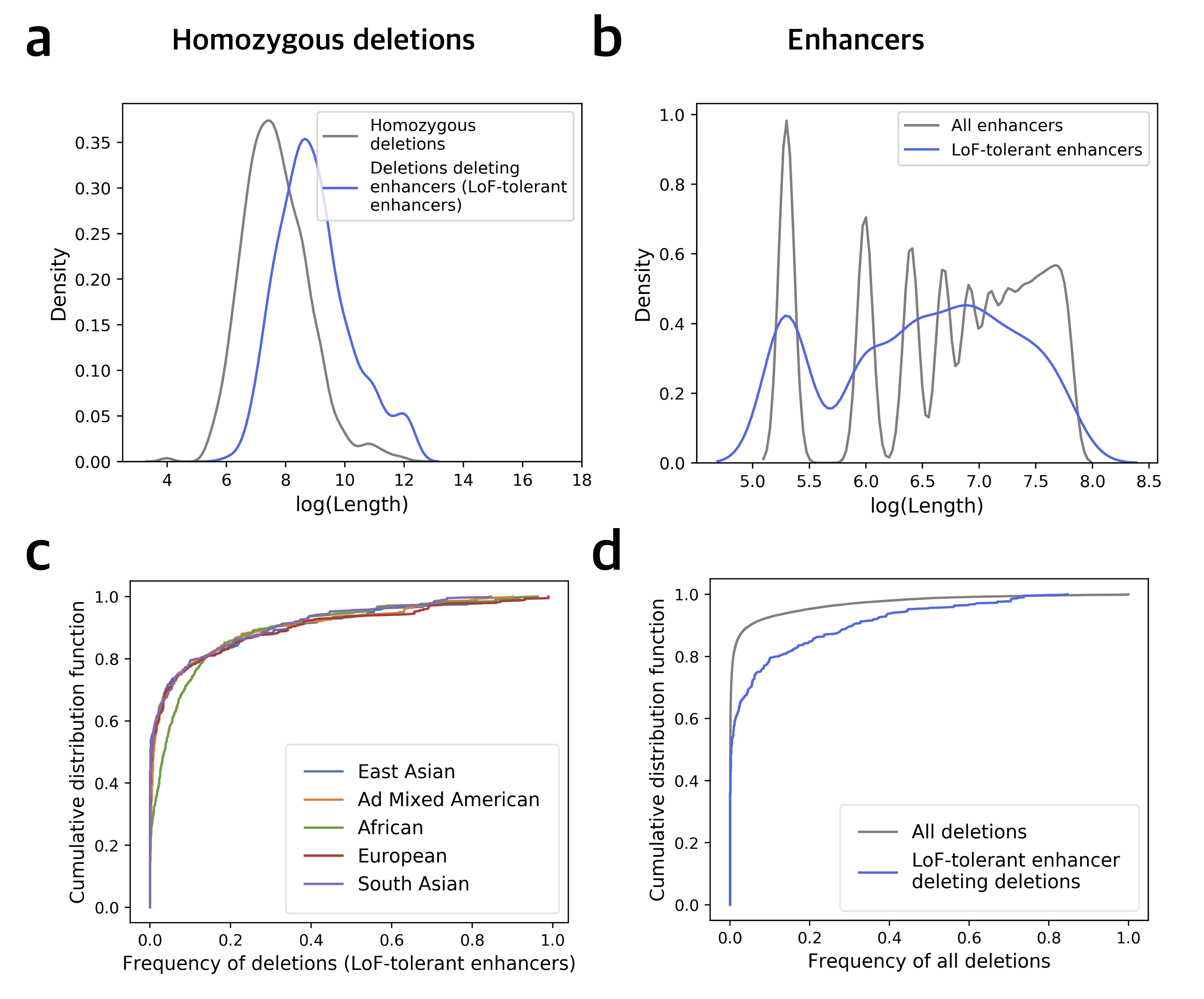

Supplement: S6 Fig — a) Length distribution of homozygous deletions that do not overlap with exons, blue marks the deletions deleting enhancers; b) Length distribution of deleted enhancers (LoF-tolerant enhancers) and all enhancers; c) Density of allele frequency of enhancer-deleting deletions by super populations (LoF-tolerant enhancers). The frequency distributions are significantly different for pair-wise comparisons of the super populations (KS, Kolmogorov–Smirnov test P-value < 0.05) except for comparison between European and South Asian. Allele frequency of LoF-tolerant enhancers are significantly higher in African population which is consistent with allele frequency distribution of all deletions in human genomes; d) Density of allele frequency of all deletions and LoF-tolerant enhancer-deleting deletions among all 1000 Genomes samples. Allele frequencies of LoF-tolerant enhancer-deleting deletions are significantly higher than all, indicating that they are more common in the population (KS test P-value = 8.33e-254). (TIF) [file pgen.1008663.s006.tif]

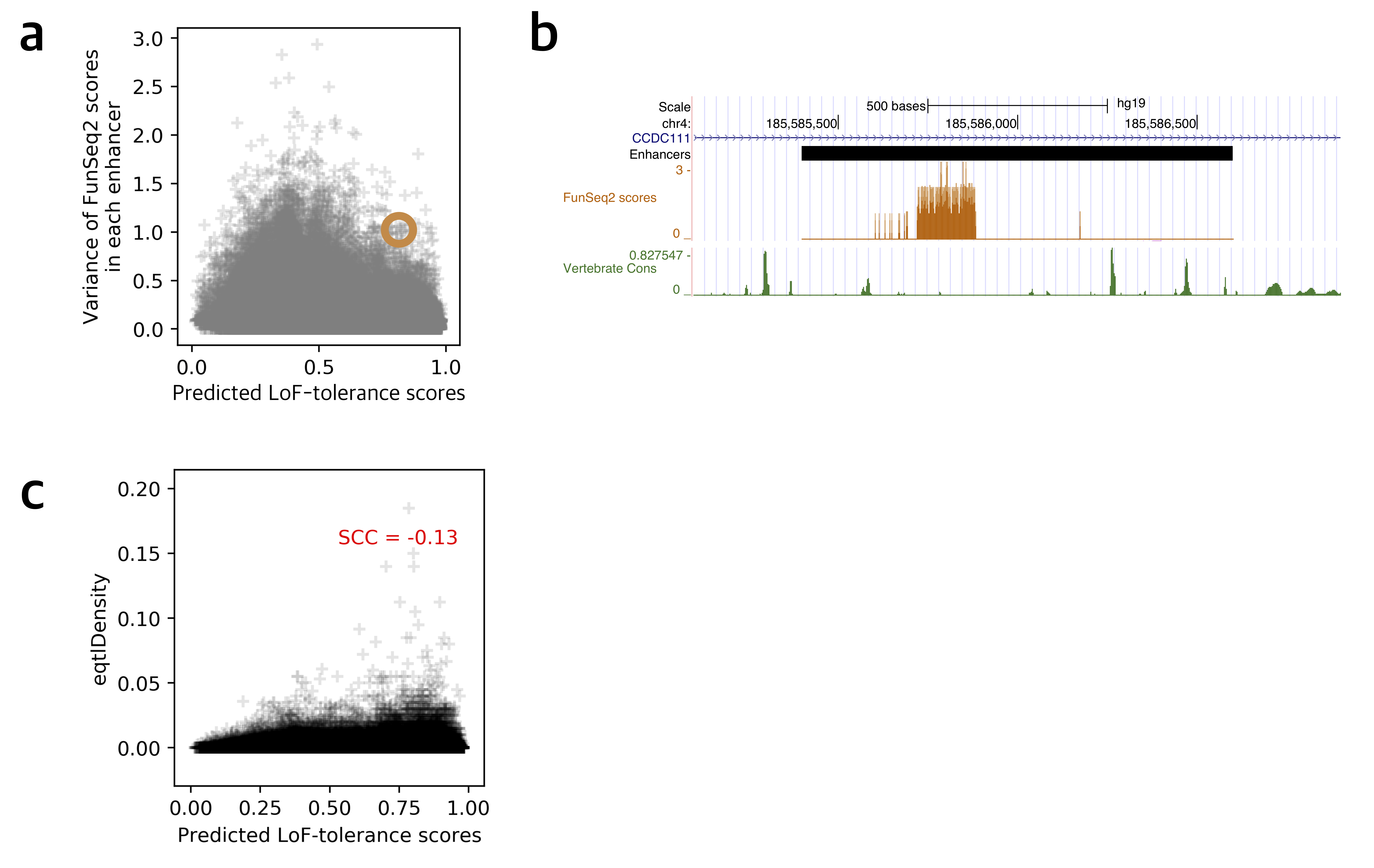

Supplement: S7 Fig — Variation of FunSeq2 scores: a) Variance of FunSeq2 scores for single nucleotide variants in each enhancer with its predicted LoF-tolerance score. Orange circle indicates the enhancer chosen for exhibition in sub-figure b; b) The genomic location of the example enhancer (chr4:185,585,400–185,586,600) with FunSeq2 scores and conservation accordingly. The example enhancer locates within an intron of CCDC111 gene and was predicted to be a LoF-tolerant enhancer with a LoF-tolerance score of 0.82. The FunSeq2 scores for mutations in this enhancer range from 0.011 (low functional impact) to 3.34 (high functional impact). The high LoF-tolerance score shows that even if a high functional impact mutation disrupts this enhancer, it will likely be well tolerated and not lead to major fitness defects. c) We found weak negative correlation between eQTL density and our predicted LoF-tolerance scores (SCC = -0.13, P-value < 2.22e-308). This is consistent with our understanding that high density of eQTLs points towards functional importance which corresponds to low LoF-tolerance scores. (TIF) [file pgen.1008663.s007.tif]

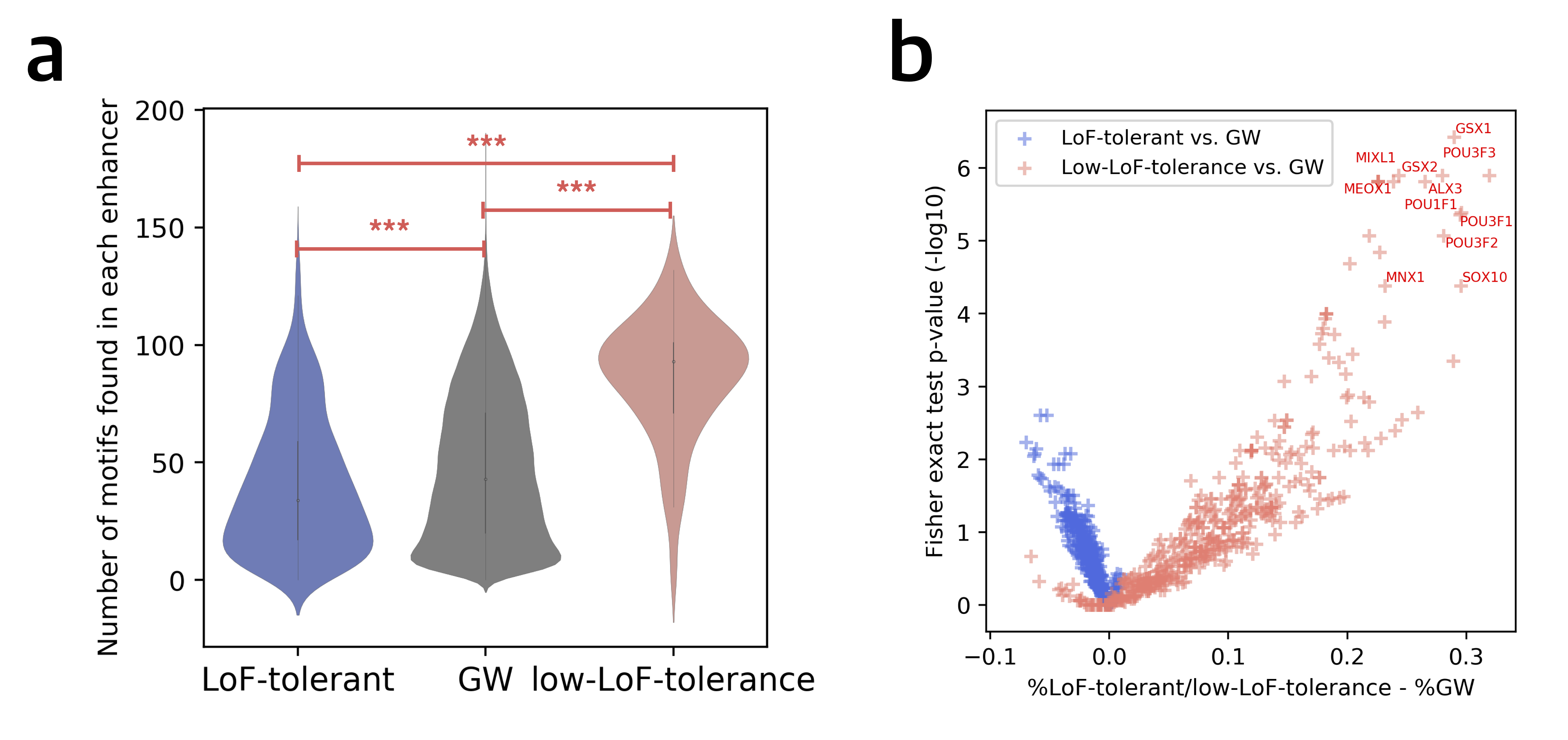

Supplement: S8 Fig — a) The y-axis shows the number of motifs found in each enhancer. The significant comparisons are marked by asterisks (Wilcoxon rank sum test P-value = 7.14e-11, 1.32e-15 and 9.22e-19 for LoF-tolerant vs. GW, low-LoF-tolerance vs. GW and LoF-tolerant vs. low-LoF-tolerance respectively); b) X-axis shows the motif presence percentage difference between LoF-tolerant vs. GW and low-LoF-tolerance vs. GW. The top 10 significantly enriched TFs in low-LoF-tolerance enhancers (adjusted Fisher exact test P-value < 0.0001) are labeled in red. (TIF) [file pgen.1008663.s008.tif]
